# Overexpression of HOXA10 is associated with unfavorable prognosis of acute myeloid leukemia

**DOI:** 10.1186/s12885-020-07088-6

**Published:** 2020-06-22

**Authors:** Chao Guo, Qian-qian Ju, Chun-xia Zhang, Ming Gong, Zhen-ling Li, Ya-yue Gao

**Affiliations:** Department of Hematology, China-Japan Friendship Hospital, Yinghua East Street, Beijing, China

**Keywords:** Acute myeloid leukemia, Survival analysis, Gene expression profiling

## Abstract

**Background:**

HOXA family genes were crucial transcription factors involving cell proliferation and apoptosis. While few studies have focused on HOXA10 in AML. We aimed to investigate the prognostic significance of HOXA10.

**Methods:**

We downloaded datasets from GEO and BeatAML database, to compare HOXA expression level between AML patients and controls. Kaplan-Meier curves were used to estimate the impact of HOXA10 expression on AML survival. The differentially expressed genes, miRNAs, lncRNAs and methylated regions between HOXA10-high and -low groups were obtained using R (version 3.6.0). Accordingly, the gene set enrichment analysis (GSEA) was accomplished using MSigDB database. Moreover, the regulatory TFs/microRNAs/lncRNAs of HOXA10 were identified. A LASSO-Cox model fitted OS to clinical and HOXA10-associated genetic variables by glmnet package.

**Results:**

HOXA10 was overexpressed in AML patients than that in controls. The HOXA10-high group is significantly associated with shorter OS and DFS. A total of 1219 DEGs, 131 DEmiRs, 282 DElncRs were identified to be associated with HOXA10. GSEA revealed that 12 suppressed and 3 activated pathways in HOXA10-high group. Furthermore, the integrated regulatory network targeting HOXA10 was established. The LASSO-Cox model fitted OS to AML-survival risk scores, which included age, race, molecular risk, expression of IKZF2/LINC00649/LINC00839/FENDRR and has-miR-424-5p. The time dependent ROC indicated a satisfying AUC (1-year AUC 0.839, 3-year AUC 0.871 and 5-year AUC 0.813).

**Conclusions:**

Our study identified HOXA10 overexpression as an adverse prognostic factor for AML. The LASSO-COX regression analysis revealed novel prediction model of OS with superior diagnostic utility.

## Background

Acute myeloid leukemia (AML) accounts for 80% of acute leukemia in adults. AML is characterized by unlimited clonal proliferation and accumulation of myeloid progenitors [1]. The 5-year survival for AML patients is no more than 50%, which is less than 20% in elderly AML patients [2]. To estimate risk and survival of AML patients, quite a few prediction models have been developed. European LeukemiaNet (ELN) 2017 risk stratification is the most commonly used risk model, which stratified AML patients based on recurrent cytogenetics and molecular mutations abnormalities [3]. A comprehensive evaluation of genetic variables is crucial for risk stratification and will guide treatment decisions. Other traditional prognostic factors include age, WBC count, LDH level etc. [4]. Whereas the biomarkers with prognostic value are still being explored to improve the risk model for AML.

The genetic alterations of transcription factors (TFs) occur frequently in AML, which exert important effects in pathogenetic process and associate with prognosis [5–8]. HOXA (homeobox protein HOX cluster A) family genes, encoding highly conserve TFs with DNA binding homeobox binding motifs, play a crucial role in adult hematopoiesis [9–11], while aberrant overexpression of HOXA promotes oncogenesis [12]. The previous studies indicated that genetic alterations in AML resulted in HOXA overexpression, such as KMT2A rearrangement [13], FLT-ITD [13], MLL gene abnormality [14–17]. HOXA9, HOXA7, HOXA11 were associated with adverse prognosis in AML [18, 19]. Overexpression of HOXA10 was reported to interrupt hematopoietic process [20] and lead to myeloid leukemogenesis in mice model [21, 22]. While the prognostic significance of HOXA10 for AML has been rarely explored.

Nowadays, multidimensional information has been accumulated for AML other than gene mutations and karyotypes, including gene expression, non-coding RNA profile, gene methylation profile, copy number variation, etc. In the present study, we explored regulatory genetic or epigenetic variables of HOXA10, such as methylation of CpG, copy number variation (CNV), lncRNA, microRNA and TF, which affect the gene expression. The interaction of lncRNA and miRNA sponges form competing endogenous RNA (ceRNA) network regulating gene expression and pathways. Then Lasso-Cox model was used to fit AML survival to prediction model, including clinical features and HOXA10-associated genetic/epigenetic variables. Our work offered evidence for using HOXA10 as a prognostic marker for AML, and establishment of novel risk model to predict AML survival.

## Methods

### Data source

We downloaded the microarray data of GSE15061 [23] (202 AML and 69 controls, Affymetrix U133 Plus 2.0 Array), GSE30029 [24] (46 AML and 31 controls, Illumina HumanHT-12 V3.0 beadchip), GSE114868 [25] (194 AML and 20 controls, Affymetrix Transcriptome Array 2.0), GSE13159 [26] (501 AML and 73 controls, Affymetrix U133 Plus 2.0 Array) form GEO database (https://www.ncbi.nlm.nih.gov/geo/). The RNA-seq data was obtained from BeatAML database [27] (474 AML and 33 controls, http,//www.vizome.org/aml). For micro-array data, expression level of a gene was calculated as the mean value (M value) of all probe sets annotating to it. For RNA-seq data from BeatAML database, the log2 transformed Reads Per Kilobase Million (RPKM) data was utilized.

### Comparison of HOXA10 expression level between AML patients and controls

The 5 public micro-array/RNA-seq datasets were used to compare HOXA10 expression level between AML patients and controls. GSE30029 dataset sampled from CD34+ bone marrow cells, while other datasets were obtained from unsorted bone marrow cells. The micro-array and RNAseq data were normalized before analysis by R program (3.6.0). Then expression level of HOXA10 was compared between AML and controls, using unpaired t-test.

### Kaplan-Meier analysis of HOXA10 on AML survival

The RNAseq data (count) of 151 AML patients was download for AML cohort from TCGA database (https://portal.gdc.cancer.gov/). The median RPKM was employed to divide the patients into HOXA10-high and HOXA10-low groups. Kaplan-Meier plot and logrank test were used to compare OS and DFS between 2 groups.

### Genome-wide gene/miRNA/lncRNA expression profiles associated with HOXA10

The differentially expressed genes/miRNAs/lncRNAs (DEGs/DEmiRs/DElncRs), between HOXA10-high and -low groups, were identified by “DESeq2” package and R (version 3.6.0). Then we accessed the HOXA10-associated cell signaling pathways using gene set enrichment analysis (GSEA) based on MSigDB database (http://software.broadinstitute.org/gsea/msigdb) [28–30].

### Overrepresentation analysis of aberrantly expressed genes associated with HOXA10

To demonstrate the implication of DEGs, the ClueGO plugin of Cytoscape software (version 3.7.3) was employed to perform functional enrichment analysis based on the Kyoto Encyclopedia of Genes and Genomes (KEGG) and Reactome pathway databases. Gene ontology (GO) based functional enrichment was conducted by the “topGO” package (R 3.6.0, Bioconductor 3.10), and summarized by the “REVIGO” package. The enriched GO/KEGG/REACTOME terms or pathways were defined to be significant with an adjusted *p* value < 0.05.

### Establishment of upstream regulatory network

The upstream TFs/miRNAs/lncRNAs, targeting HOXA10 gene, were identified, based on TF/microRNA/lncRNA target predicting algorithm and correlating analysis, using Gene Transcription Regulation Database (GTRD, http://gtrd.biouml.org/) [31], miRWalk 2.0 (http://zmf.umm.uni-heidelberg.de/) [30, 32] and prediction module of lncBase v2 [33] online tools. Then combined with the results of genome-wide expression analysis, we identified the overlapped genes/miRNAs/lncRNAs, which were predicted to target HOXA10 and differentially expressed.

### Establishment of prediction model for AML survival

In order to improve the prognostic model, a comprehensive survival analysis was performed, which integrated clinical features, HOXA10 expression, methylation, CNV and upstream TFs/microRNAs/lncRNAs expression. RNA-seq data (counts, IlluminaHiSeq), miRNAseq data (RPM value, illuminaGA), beta value of methylation (HumanMethylation450), and gene-level copy number data (GISTIC 2 method) regarding HOXA10 were downloaded from the TCGA database (https://portal.gdc.cancer.gov/). Since AML-M3 patients have a distinct prognostic profile in comparison with other subtypes, we excluded such patients. The traditional prognostic factors were brought into analysis, including age, gender, race, risk stratification of cytogenetics, risk stratification of molecular mutations and WBC count. The molecular mutations and cytogenetic risk stratification were based on ELN2017 recommendations [3], which classified AML patients into good/intermediate/poor groups based on molecular mutations and cytogenetics. Finally, we screened and included113 AML patients with all abovementioned information. The OS and included factors were fitted to least absolute shrinkage and selection operator (LASSO) -Cox model, resulting in prediction model for AML survival.

### Statistical analysis

We established a LASSO-Cox regression model [34], using glmnet package and R program (3.6.0). LASSO method for variable selection penalizes the data fitting criteria, which gets rid of less informative predicting variables to reduce complexity and maker models more interpretable. For each of the LASSO-screened variables, the final coefficients were the average estimates of the coefficients obtained from cross-validation development. To evaluate the diagnostic utility of prediction model, time dependent receiver operating characteristic (ROC) curve was used and 1-year/3-year/5 year area under curve (AUC) were calculated. Wilcoxon rank-sum was employed for comparisons of continuous variables between subgroups. Chi-square tests were used to test the association of categorical variables.

## Results

### Overexpression of HOXA10 in AML

**Fig. 1:**
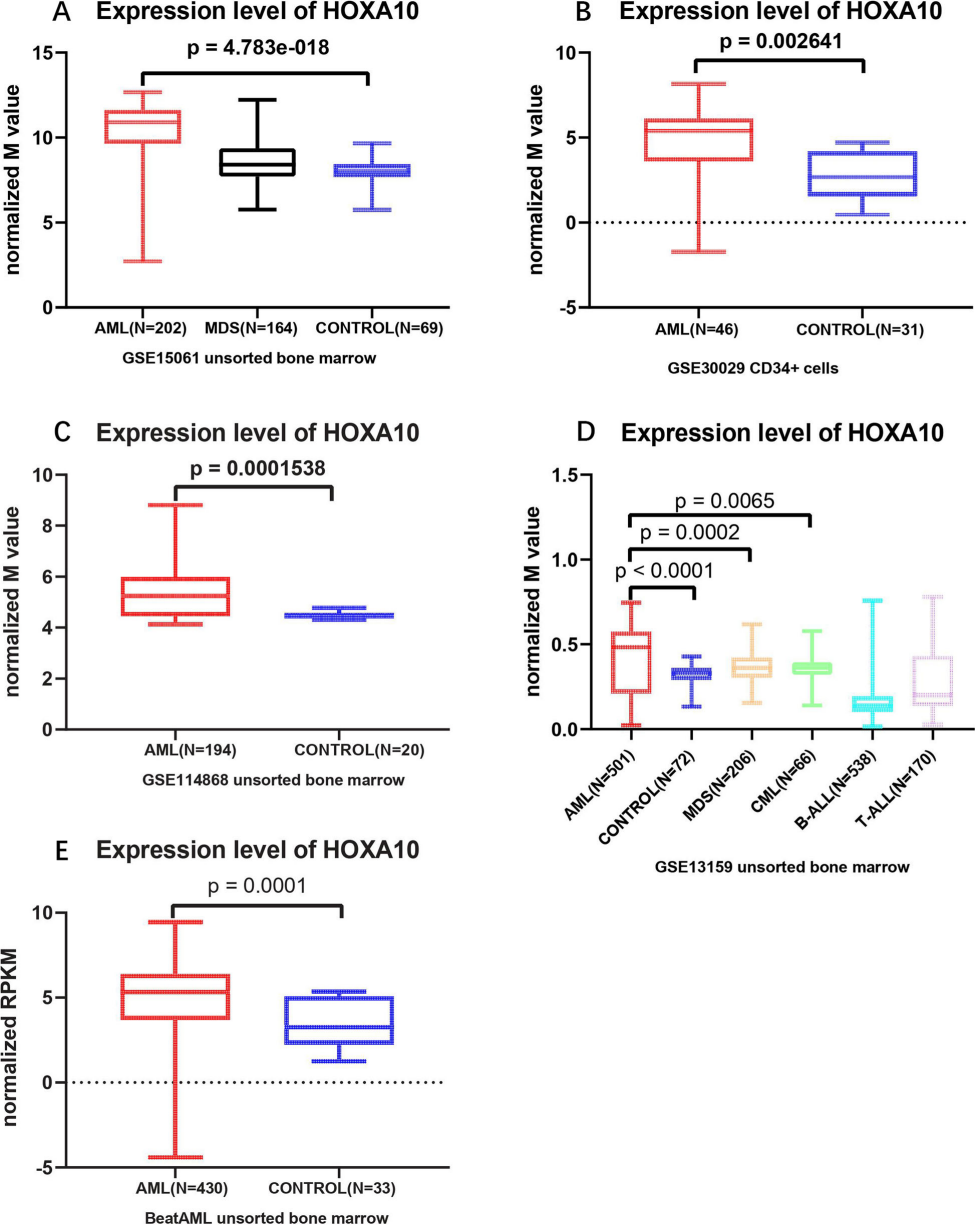
Comparison of expression level in AML patients and controls using data derived from GSE15061(**a**), GSE30029(**b**), GSE114868(**c**), GSE13159(**d**) and BeatAML(**e**). The expression level of HOXA10 was represented by log2 transformed normalized mean (M) value or RPKM value. Underlying difference of HOXA10 expression was revealed between AML and healthy control group by all datasets (**a**-**e**), and the difference between AML and other myeloid malignancy (MDS, CML) was uncovered by GSE13159 (Fig. **d**). The conclusion was robust no matter the expression data was derived from unsorted bone marrow or CD34+ cells

The higher expression level of HOXA10 was revealed in AML patients than that in control group, in unsorted and CD34+ bone marrow cells (Fig. 1a-e). HOXA10 expression signature was similar across different cell subpopulations of AML. Furthermore, results from GSE13159 implicated that the HOXA10 expression was higher in AML than that in other myeloid neoplasms (MDS/CML) and lymphoid malignancies (T-ALL/B-ALL), which indicated that HOXA10 overexpression may be AML-specific signature.

### High expression of HOXA10 is associated with adverse prognosis

The clinical characteristics of HOXA10-high and -low groups were shown in Table 1. The proportion of AML-M3 is higher in HOXA10-low group than that in HOXA10-high group (*p* < 0.0001). HOXA10-high AML patients were associated more advanced ELN2017 risk stratification (p < 0.0001).

**Table 1:** The comparison of clinical and genetic features between HOXA10-high and HOXA10-low groups

	HOXA10-low group (*n* = 72)	HOXA10-high group (*n* = 73)	*p* value
**Age (year)**	52.08 ± 17.592	56.63 ± 14.730	0.094
**Gender**			0.739
Female	31	34	
Male	41	39	
**Race**			0.717
White	50	53	
Other races	22	20	
**Mutation count**	9.43 ± 5.897	9.63 ± 5.116	0.826
**FAB subtype**			< 0.0001
M3	15	0	
non-M3	57	73	
**Risk stratification of cytogenetics**			< 0.0001
Good	29	1	
Intermediate	26	55	
Poor	16	15	
**Risk stratification of molecular mutation**			< 0.0001
Good	30	1	
Intermediate	25	50	
Poor	16	20	
**WBC**	28.693 ± 45.009	40.800 ± 36.862	0.078

**Fig. 2:**
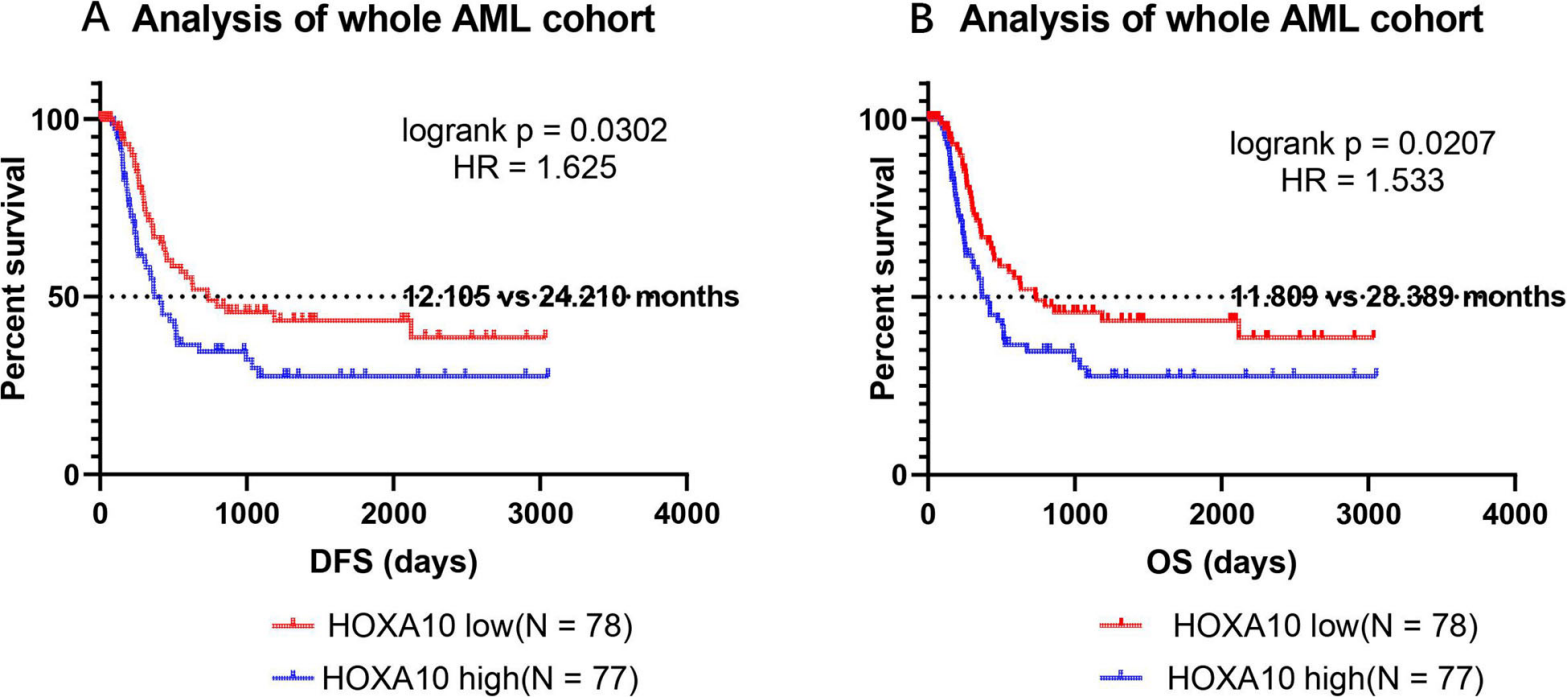
The Kaplan-Meier plots of HOXA10 expression for DFS (**a**) and OS (**b**). The survival (DFS and OS) of HOXA10-low AML group is twice as longer as that of HOXA10-high group

The Kaplan-Meier plots indicated that the AML survival of HOXA10-high group was significantly shorter than that of HOXA10-low group (Fig. 2a&b). Median OS of HOXA10-high and -low groups are 12.105 vs 24.210 months respectively, and logrank *p* value is 0.0302. The median DFS of HOXA10-high and low are 11.809 vs 28.389 months respectively, and logrank p value is 0.0207. The results indicated that HOXA10 overexpression is an adverse prognostic factor for AML patients.

### Genome-wide gene/miRNA/lncRNA profiles associated with HOXA10 expression

**Fig. 3:**
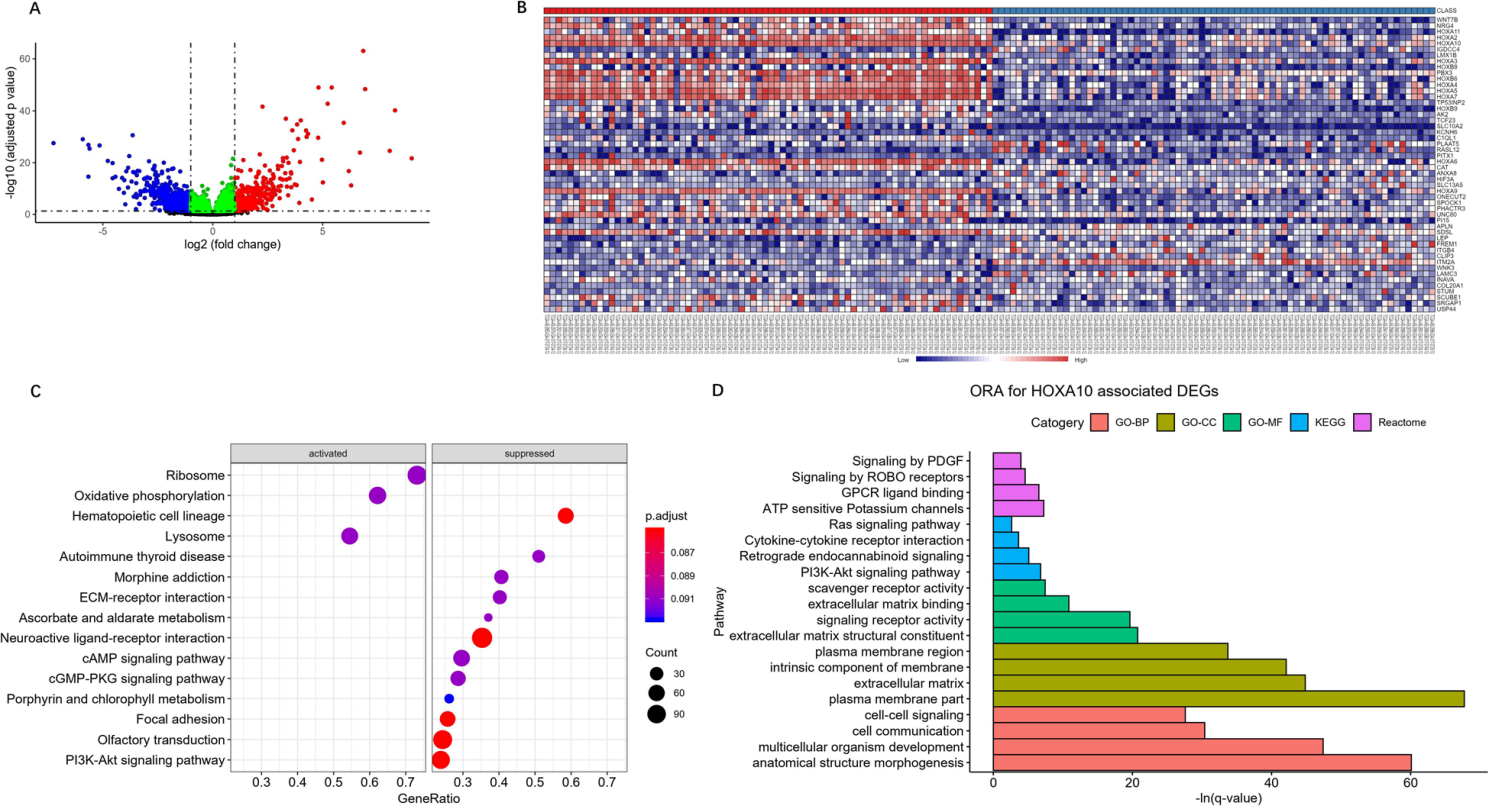
The volcano plot (**a**) and heatmap (**b**) showed the distribution and top DEGs. 1219 DEGs were reveal by comparing the gene expression level between HOXA10-high and low group, with 433 upregulated DEGs and 786 downregulated DEGs. The heatmap showed the distinct expression signature of HOXA10-high (left columns) and low groups (right columns), displaying top differentially expressed genes screened by adjusted *P* value. The over-expressed genes were colored in red, while the under-expressed genes were in blue. The dotplot of GSEA results and barplot of ORA results were shown in c and d, respectively. In **c**, the significantly activated and suppressed pathways derived from GSEA were listed, in which the color of dots represent the adjusted P value and the diameter stand for enriched gene count. In **d**, the top enriched pathways obtained from GO/KEGG/Reactome analysis were shown, while the X axis stands for -ln(q value)

1219 DEGs, 131 DEmiRs and 282 DElncRs were identified by comparing HOXA10-high and HOXA10-low AML groups from TCGA (Fig. 3a). The heatmap for top DEGs was shown in Fig. 3b, in which the top differentially expressed genes, filtered by adjusted p value, were revealed between HOXA10-high and low groups. Wnt Family Member 7B (WNT7B), Neuregulin 4 (NRG4) and HOXA11 were the top DEG, which were overexpressed in HOXA10-high groups in comparison with that in HOXA10-low group. Other HOX family genes, like HOXA2/HOXA3/HOXA4/HOXA5/HOXA6/HOXA7/HOXB6/HOXB8/HOXB9, were upregulated correlating to HOXA10 expression. The distinct expression signature between 2 groups may help us to investigate and uncover potential biomarkers.

The results of GSEA indicated 12 suppressed cell pathways and 3 activated pathways were significantly correlated with HOXA10 expression (Fig. 3c & Supplementary Table 1). The suppressed pathways in HOXA10-overexpressed patients included PI3K-Akt signaling, hematopoietic cell lineage, cAMP signaling, etc. while the activated pathways included ribosome, oxidative phosphorylation, lysosome, etc.

### Overrepresentation analysis for DEGs

GO analysis revealed that DEGs were significantly enriched in the following biological processes (BP): cell-cell signaling, cell communication, etc. Cell component (CC) analysis revealed that DEGs were predominantly located in the plasma membrane region, extracellular matrix, etc. Molecular mutations function (MF) analysis demonstrated that DEGs were enriched in signaling receptor activity, extracellular matrix structural constituent, etc. For KEGG pathway analysis, DEGs were significantly enriched in the following pathways: PI3K-Akt signaling pathway, Ras signaling pathway, etc. In Reactome analysis, the DEGs were enriched in signaling by PDGF, signaling by ROBO receptor, etc. The detail ORA (overrepresentation analysis) results are listed in Supplementary Table 2, and top enriched pathways are shown in Fig. 3d.

### Genome-wide methylation profile associated with HOXA10 expression

A total of 76 DMRs within exonic regions were uncovered. The detail DMRs within exonic regions were listed in Supplementary Table 3. The methylation level of HOXA10 was significantly negatively correlated with HOXA expression. Among the methylated CpG sites of HOXA10, cg21172377 is significantly differentially methylated along with HOXA10 expression. And the AML patients with hypermethylated cg21172377 have significantly shorter OS, according to survival analysis using MethSurv [35] (https://biit.cs.ut.ee/methsurv/). Therefore, beta value of cg21172377 was used to represent the methylation level of HOXA10 in the following analysis.

### Regulatory network of HOXA10

**Fig. 4:**
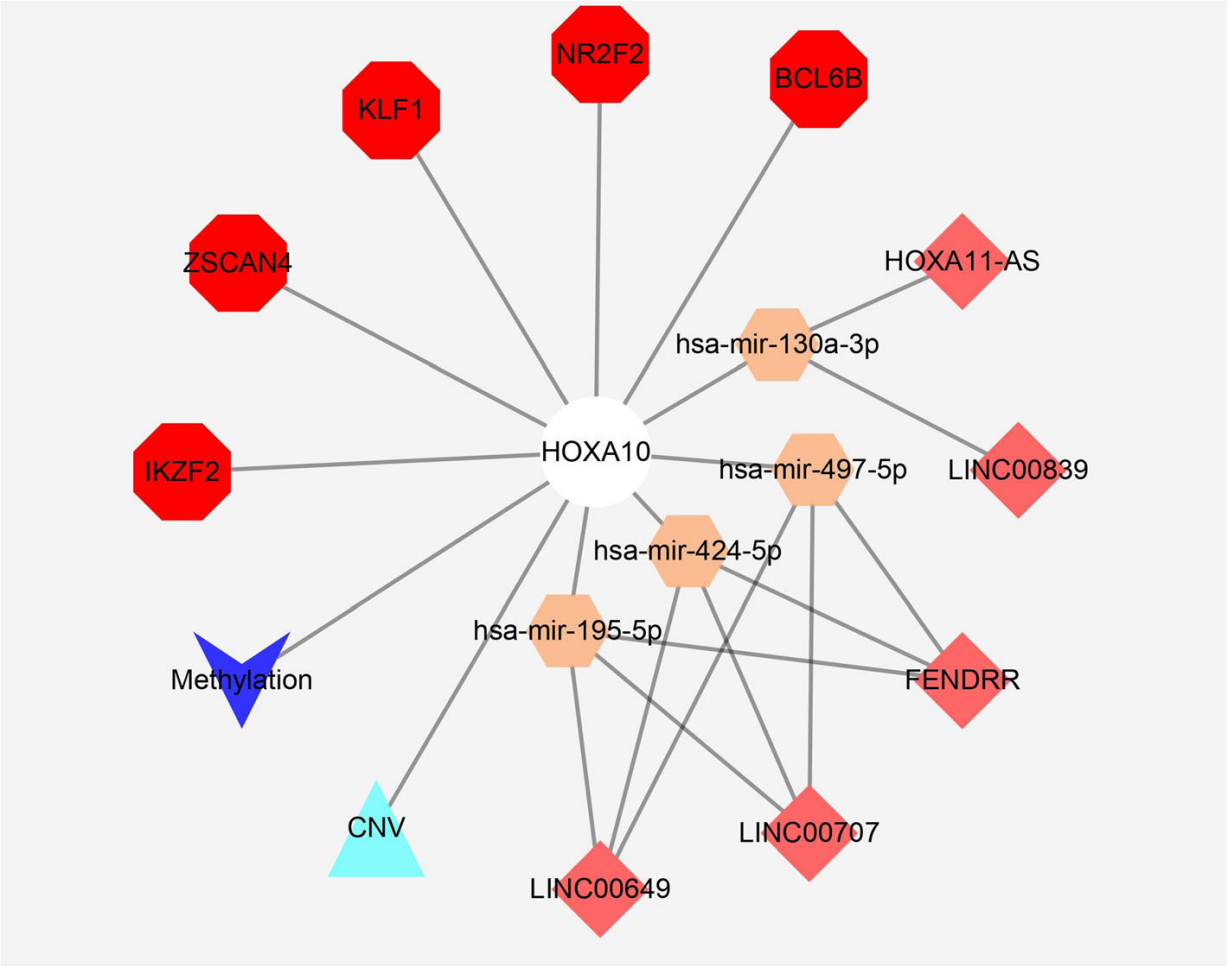
The regulatory network of HOXA10 expression consisting related TFs, lncRNAs, microRNAs, which were then inputted as variables in LASSO analysis to reveal the prediction model

The upstream ceRNA network and TFs regulating HOXA10 were shown in Fig. 4. The HOXA10 related TFs/miRNAs/lncRNAs included BCL6B, NR2F2, KLF1, ZSCAN4, IKZF2, LINC00649, LINC00839, LINC00707, HOXA11-AS, FENDRR, miR-424-5p, miR-130a-3p, miR-497-5p and miR-195-5p, all of which are predicted by miRWalk 2.0 and lncBase v2 online tools and differentially expressed between HOXA10-high and -low group.

### Identification of key prognostic markers

**Fig. 5:**
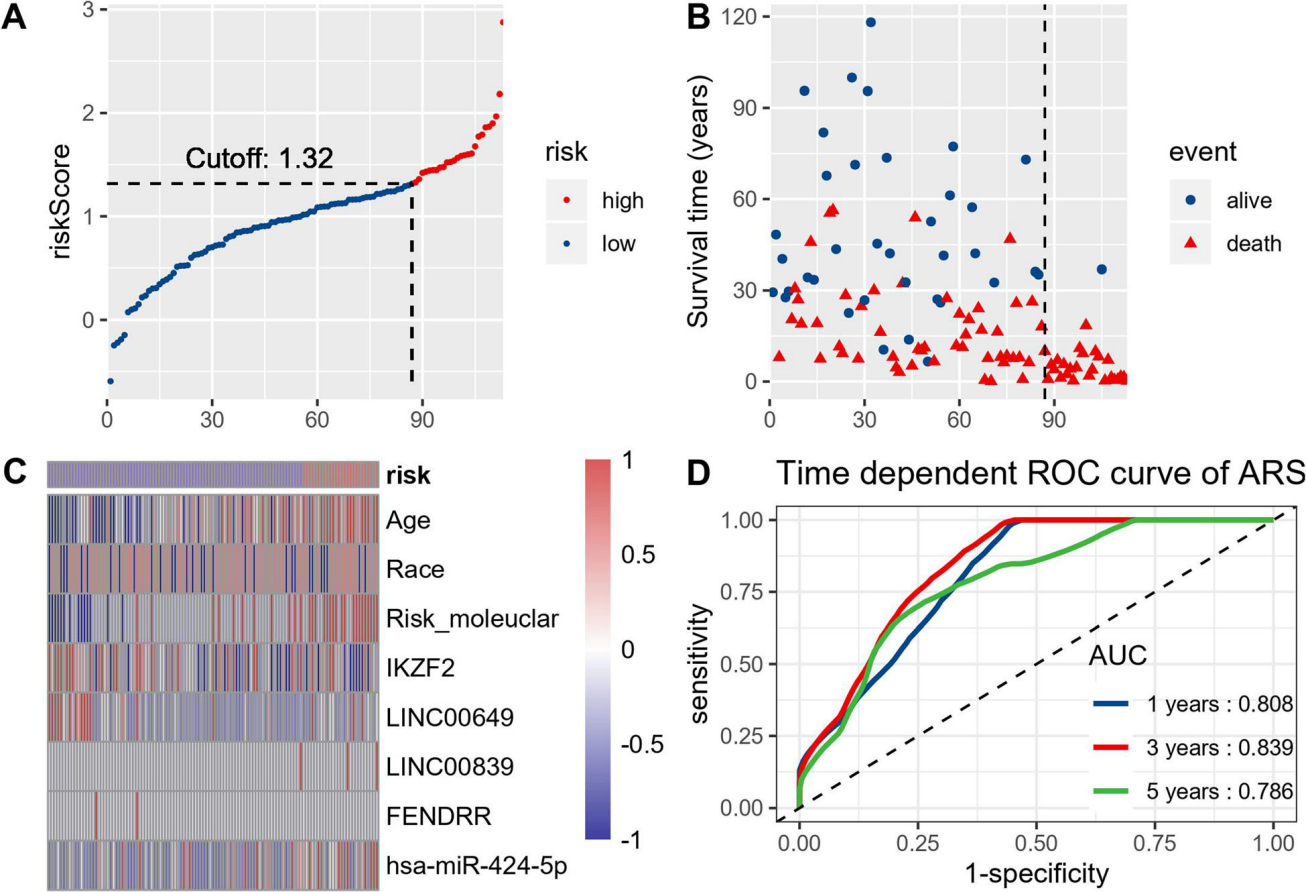
**a** showed the distribution of risk score calculated by ARS model in 113 AML patients, with 1.32 as the cutoff value. **b** displayed the survival events distribution, in which the dotted line separated the high and low risk groups. Risk to variable heatmap (**c**) revealed the top variables that distributed differently between high and low risk group. Time dependent ROC curves of ARS model showing 1-year/3-year/5-year AUC in **c**, showing the diagnostic utility

LASSO regression analysis identified the OS fitted predictive model, including age, race, molecular risk, expression level of IKZF2/LINC00649/LINC00839/FENDRR/has-miR-424-5p. The developing model process identified AML-survival risk scores (ARS) to calculate each patient using variables weighted by coefficients (Table 2). The cut-off value was calculated by “cutoffROC” package (Fig. 5d), which is 1.315. Time dependent ROC was performed using survivalROC package, and the 1-year/3-year/5-year AUC are 0.808/0.839/0.786 respectively, implicating the diagnostic utility is satisfying.

**Table 2:** The variables and coefficients of ARS model

Variable	Coefficients
age	1.56E-02
race	5.58E-02
risk_molecular	4.95E-01
IKZF2	−6.49E-02
linc00649	−1.71E-05
linc00839	9.02E-05
FENDRR	−1.33E-05
has-miR-424-5p	3.32E-04

**Fig. 6:**
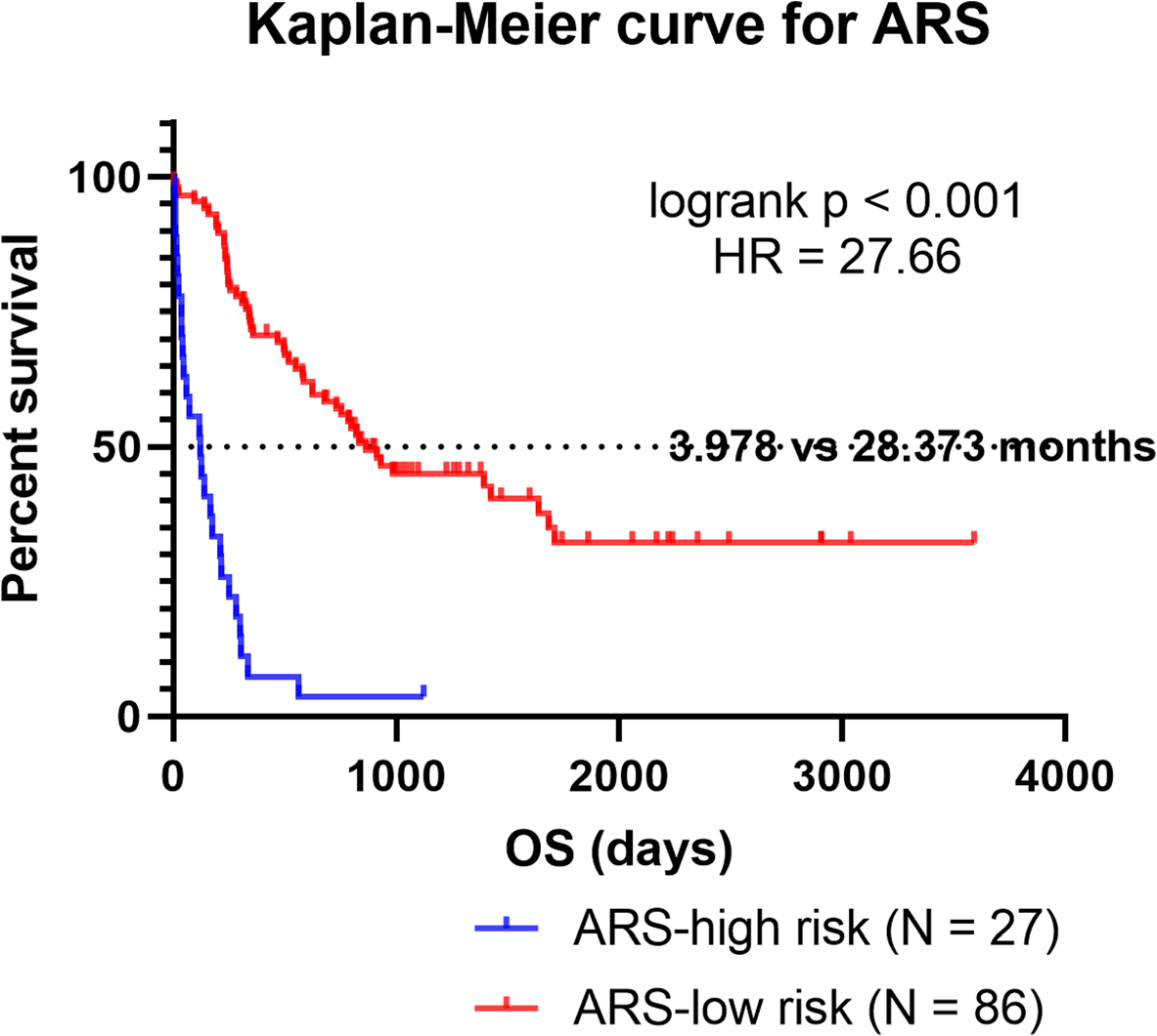
The Kaplan-Meier plot comparing the overall survival of ARS-high and ARS-low groups. Notably, the survival of ARS-high group is far more inferior

By the cut-off ARS value, the AML cohort was classified as high-risk and low-risk groups. The ARS distribution plot, survival-events plot and risk-to-variable heatmap were shown in Fig. 5a&b&c. Then we used Kaplan-Meier plot to analysis the survival difference between ARS-high risk and low risk group (Fig. 6, logrank *p* < 0.0001, HR = 27.66). The median OS of ARS-low patients is 28.373 months and that of ARS high patients is only 3.987 months. The performance of ARS model is encouraging, but further prospective studies are needed to evaluate the predictive value of this model.

## Discussion

HOXA family genes are well known as the crucial transcription factors in pathogenesis and development of AML. HOXA10 belongs to the HOXA gene superfamily, dysregulation of which has been observed in several solid tumors [36–38]. HOXA10 plays a role in myeloid differentiation, leukemogenesis and chemoresistance in AML [39–41]. In the present study, we investigated the prognostic significance of HOXA10 in AML, which has been rarely described and reported previously. The results of expression analysis demonstrated that AML patients have aberrant HOXA10 expression in comparison with controls. High HOXA10 expression level is significantly associated with worse OS and DFS of AML, based on Kaplan-Meier curve and logrank test. Therefore, HOXA10 may serve as a prognostic marker for AML patients.

To explore the underlying enriched pathways of DEG, the ORA was performed. In the GO part of our ORA, we uncovered that the biological processes of DEG were enriched in cell-cell signaling and cell communication, and the cell components were enriched in plasma cell membrane and extracellular matrix. Correspondingly, the molecular functions were enriched in signaling receptor activity and extracellular matrix structural constituent. To explore the detail cell-cell signaling pathway that the DEGs were involved, the following KEGG and Reactome analysis revealed that the DEGs were enriched in PI3K-Akt signaling, Ras signaling pathway, signaling by PDGF and signaling by ROBO receptor, etc. In addition to activating mutation of NRAS/KRAS in 15–25% AML, the mutations of RAS-regulating genes (NF1 and PTPN11) and RAS-signaling receptor (FLT3 and KIT) are also harbored in AML frequently [42–46]. Hyperactive mutations of NF1 and PTPN11 gene are associated with inferior survival in pediatric and elderly AML [47, 48]. FLT3-ITD mutation is also well-known as a biomarker for worse prognosis in non-APL AML patients [49, 50]. Although NRAS and KRAS genes was not significantly differentially expressed, RAS signaling genes, including PTPN11, FLT3 and KIT, were upregulated significantly in HOXA10-high AML patients. The dysregulation of RAS signaling pathway may lead to unfavorable impact on clinical outcome of HOXA10-high group. PDGF signaling plays a proto-oncogenic role in diverse cancer cells. Imatinib turned out to block PDGF receptor at low dose, exerting a pharmacological effect for BCR-ABL positive CML and FIP1L1-PDGFRA mutated eosinophilic leukemia patients [51, 52], suggesting PDGF signaling as an activated effector in hematological malignancies. Gołos A et al. reported increased ROBO1/2 in AML patients in comparison with normal control, and high ROBO3 expression is associated with cytogenetic high risk and poor prognosis [53]. However, the aberrantly expression pattern of signaling pathway by PDGF/ROBO has not been fully elucidated in AML, which were rarely studied in the prognosis of AML. The enriched pathways obtained by ORA help us to identify expression signature in HOXA10-high group, screen useful biomarkers and provided novel insights into molecular investigation on AML.

Differential expression analysis and GSEA revealed that PI3K-Akt signaling pathway was suppressed associated with HOXA10 overexpression. PI3K-Akt signaling pathway is frequently activated in AML, constitutive activation of PI3K and Akt were found in 50% de novo AML patients [54, 55]. The PI3K-Akt signaling controls leukemic blast cells proliferation and clonogenicity [56, 57]. AML patients with constitutive PI3K-Akt activation have better OS and relapse-free survival [58]. The unfavorable survival profile in HOXA10-high group may attributed to aberrant downregulation of PI3K-Akt signaling.

The results of GSEA demonstrated that ribosome, oxidative phosphorylation, and lysosome pathways were activated in HOXA10-high group. Ribosome pathway is a vital cellular process, and the rate-limiting step of which is the initiation of translation in ribosomes. One of major control factors in the ribosome activity, is EIF2 (eukaryotic initiation factor 2), which is regulated by phosphorylation of α subunit (EIF2α) under diverse stress. Four kinds of EIF2α kinases (EIF2AK1/2/3/4) can affect the activity of EIF2α by phosphorylation of Ser51 [59, 60]. Notably, the expression EIFAK2 and EIFK3 were significantly increased in HOXA10-high group by our results. EIF2AK2 (also named as double-strand RNA-dependent kinase, PKR) responses to various types of stress, including DNA damage, mitochondrial stress, viral infection, growth factor deprivation, cytokines, Toll-like receptor activation and cytotoxic drugs [61–65]. Also EIF2AK2 is the only EIF2α kinase exists in both the cytoplasm and nucleolus, while other 3 kinases present only in the cytoplasm [66]. Cheng X et al. reported that high expression of EIF2AK2 was associated with worse prognosis in AML, and it reduced DNA damage response by inhibiting ataxia-telangiectasia mutated (ATM) activation, leading to accretion of leukemia in mice model [67]. EIF2AK3 (also named as PKR-like endoplasmic reticulum kinase, PERK) is reported to promoted leukemia progress by stimulating the dissemination of leukemia cells in vivo [68]. So, the increased EIF2AK2/3 expression and activated ribosome pathway contributed to the worse outcomes in HOXA10-high patients. The maintenance of leukemia stem cells depends on BCL2 mediated oxidative respiration, instead of glycolysis as in normal hematopoietic cells [69]. The metformin, targeting oxidative phosphorylation (OXPHOS), induces apoptosis of human leukemia cells in an AMPK-independent way [70]. Cytarabine resistant leukemia cells are characterized by activated OXPHOS, with the high level of reactive oxygen species. Additionally, the resistance can be reversed by agents inducing low OXPHOS status [71]. The activation of OXPHOS in HOXA10-high patients may promoted leukemia cell maintenance and chemo-resistance, leading to inferior survival. The biological function of lysosome pathway in AML has not been fully elucidated. While considering lysosome pathway involves in autophagy, which plays a role in leukemic transformation of normal hematopoietic stem cells and chemotherapy response [72], it may be still valuable to explore in this area. The Kaplan-Meier curves confirmed that HOXA10 expression is associated with AML survival, while it didn’t predict OS or DFS significantly in multivariable Cox hazards analysis including other clinical and genetic variables (data not shown), suggesting HOXA10 is not an independent prognostic factor. Since copy number variation (CNV), mutations (not reported in TCGA database), CpG methylation status and upstream TFs/microRNA/lncRNA are the most common gene expression regulators, we investigated whether these factors were associated with HOXA10 expression and established Lasso-Cox model fitting OS to reveal novel prognostic markers. And finally, Lasso-Cox analysis included clinical features, CNV of HOXA10, methylation and expression status of HOXA10, HOXA10 associated TFs/DEmiRs/DElncRs. Due to the obvious correlation and dependency between each variable, the traditional multivariable Cox analysis is of limited utility, where the Lasso-Cox methods showed its superiority. A few prediction models for AML overall survival have been reported (Table 3), including Huang R et al. [73], Mihyang Ha et al. [74], Clinseq-G model [75], ELN2017 recommendation [75], Zejuan Li et al. [75]. The AUC equals to the probability, which a diagnostic classifier will rank a randomly chosen positive instance higher than a negative one, and the highest AUC is established as best practice [76]. Our prediction model has superior AUC than above models, possibly attributing to integrated multidimensional information integrated.

**Table 3:** Diagnostic utility of prognostic models for AML by time-dependent ROC

	1-year AUC	3-year AUC	5-year AUC
Huang R et al.(75)	0.666	0.713	0.707
Mihyang Ha et al.(76)	–	–	0.613
Clinseq-G model(77)	–	0.73	–
ELN2017	–	0.65	–
Zejuan Li et al.(77)	–	0.7	–
ARS model	0.808	0.839	0.786

In the ARS model, novel markers are included. IKZF2 is recently found to drive leukemia stem cell renewal and inhibit myeloid differentiation, by regulating HOXA9 and CEBP [77]. The aberrant variation of IKZF2 was also reported in ovarian cancer cell lines [78], adult T cell leukemia [79] and gastric cancer [80], suggesting IKZF2 as a potential oncogenes. The relation of AML and LINC00649 has not been explored. While notably, the relation of HOXA family genes and LINC00649 is very close. Based on Gepia 2.0 (http://gepia2.cancer-pku.cn/), using AML RNAseq dataset of TCGA and Pearson method, LINC00649 is negatively correlated with HOXA1 (*p* = 0.015), HOXA2 (*p* = 0.0056), HOXA3 (*p* = 0.0045), HOXA4 (*p* = 0.011), HOXA5 (*p* = 0.0019), HOXA6 (*p* = 0.0078), HOXA7 (*p* = 0.00089), HOXA9 (*p* = 0.002) and HOXA10 (*p* = 0.0022) significantly. Intriguingly, expression level of all above HOXA genes are significantly associated with AML survival [8], which indicated that LINC00649 targeted genes may exert pathogenetic effect in AML. The investigation of LINC00839, FENDRR and has-miR-424-5p in AML has not been conducted.

The limitation of this model is also obvious, which is lacking prospective large-scale studies for validation. And further experiment is needed to validate the interaction of TFs/microRNAs/lncRNAs with HOXA10.

## Conclusions

We identified the overexpression of HOXA10 as an adverse prognostic factor of AML OS and DFS. The novel multidimensional prediction model was established with satisfying diagnostic utility. These results need further clinical and experimental validation.

## Supplementary information

**Additional file 1: Table S1.** GSEA results associated with HOXA10 expression derived from TCGA database.

**Additional file 2: Table S2.** ORA results of HOXA10 associated DEGs.

**Additional file 3: Table S3.** The detail list of exonic DMRs.

## Data Availability

The data that support the findings of this study are available from GEO database (https://www.ncbi.nlm.nih.gov/geo/), BeatAML database (http://www.vizome.org/aml) and TCGA database (https://portal.gdc.cancer.gov/), which are all publicly available.

## Abbreviations

AML: Acute myeloid leukemia
ELN: European LeukemiaNet
CNV: copy number variation
RPKM: Reads Per Kilobase Million
HOXA: homeobox protein HOX cluster A
ceRNA: endogenous RNA
DEGs/DEmiRs/DElncRs: differentially expressed genes/miRNAs/lncRNAs
KEGG: Kyoto Encyclopedia of Genes and Genomes
GO: Gene ontology
LASSO: least absolute shrinkage and selection operator
